# Comparison of overall survival across treatment modalities for oesophageal, gastroesophageal, and gastric cancer: protocol for a systematic review and network meta-analysis

**DOI:** 10.1186/s13643-026-03175-0

**Published:** 2026-04-14

**Authors:** Fani Apostolidou-Kiouti, Anna Bettina Haidich, Dimitris Mavridis, Aikaterini Ioanna Apostolaki, Dimitris Tsavdaris, Adriani Nikolakopoulou

**Affiliations:** 1 Department of Hygiene, Social-Preventive Medicine & Medical Statistics, School of Medicine, Faculty of Health Sciences, Aristotle University of Thessaloniki, University Campus, Thessaloniki, 54124 Greece; 2 Department of Primary Education, University of Ioannina, Ioannina, Greece; 3 Aristotle University of Thessaloniki, Thessaloniki, 54636 Greece; 4 Faculty of Medicine and Medical Center, Institute of Medical Biometry and Statistics, University of Freiburg, Freiburg, Germany

**Keywords:** Network meta-analysis, Protocol, Upper gastrointestinal tract neoplasms, Treatment modalities

## Abstract

**Background:**

Cancers of the oesophagus, gastroesophageal junction (GEJ), and proximal stomach share epidemiological, molecular, and therapeutic characteristics. Despite their similarities, treatment guidelines vary, and the classification of GEJ tumours remains debated. While multiple meta-analyses have addressed subsets of these malignancies, a comprehensive synthesis comparing all treatment modalities across this disease spectrum is lacking. This study aims to perform a systematic review and network meta-analysis (NMA) to compare the overall survival outcomes of different treatment modalities, including surgery, chemotherapy, chemoradiotherapy, immunotherapy, and multimodal approaches, in patients with oesophageal, GEJ, and gastric cancers.

**Methods and analysis:**

We will conduct a systematic literature search in MEDLINE (PubMed) and The Cochrane Library (CENTRAL) without date restrictions. Randomised controlled trials (RCTs) comparing eligible treatments will be included. The primary outcome will be overall survival, defined as time from diagnosis to death from any cause. Secondary outcomes will include progression-free survival, disease-specific survival, dropout rates, treatment-related adverse effects, patterns of relapse, R0 resection rates, surgical morbidity, and mortality. We will look for individual patient data (IPD) from primary studies’ authors and registries. We will assess risk of bias with the Cochrane risk of bias RoB2 tool. We plan to present each pairwise comparison with risk ratios and 95% confidence intervals from random-effects meta-analysis. A random-effects NMA model will simultaneously compare all treatments in the network. We will rank interventions using P-scores. In case we manage to access IPD and we believe the transitivity assumption does not hold for a specific comparison (or we have a disconnected network), we will use population adjustment methods to estimate an indirect treatment comparison. We will apply CINeMA to assess confidence in the findings and we will report results according to PRISMA-NMA.

**Discussion:**

Neoplasms of the oesophagus, the gastroesophageal junction, and the stomach are increasingly being studied together in clinical trials, a trend driven by continuous research on their molecular characteristics and shared therapeutic patterns. This NMA aims to pool evidence employing recent advances in meta-analytic models and critically assess the confidence in the results by implementing the CINeMA approach.

**Systematic review registration:**

Registered in PROSPERO (CRD42025634169).

## Background

Neoplasms of the oesophagus, the gastroesophageal junction (GEJ), and proximal gastric neoplasms share common characteristics in their epidemiology, natural history, and therapeutic strategies. Treatment guidelines have evolved [1–3] as new treatments emerged; however, observational research suggests that distinct differences may affect the effectiveness of suggested treatment strategies. The anatomical and histopathological proximity of these malignancies has been systematically researched over the years [4–6]; the classification system that would delineate carcinomas of the region as separate entities is still an open question [7]. The rising incidence of oesophageal adenocarcinoma in Western populations, often attributed to obesity, gastroesophageal reflux, and Barrett’s oesophagus, is paralleled by an increase in proximal gastric cancers, suggesting overlapping risk factors and shared disease mechanisms [8]. Cancers at the GEJ frequently display intermediate characteristics between oesophageal and gastric adenocarcinomas, making precise classification challenging. This ambiguity is reflected in the clinical management of GEJ cancers, which often follows guidelines for both oesophageal and gastric tumours [3, 8, 9].

The rationale on pooling data from oesophageal cancer, gastroesophageal junction cancer (GEJ), and gastric cancer is supported by their shared molecular, clinical, and therapeutic characteristics. Molecular analyses have shown that oesophageal adenocarcinomas and the chromosomally unstable subtype of gastric adenocarcinomas (CIN) display significant overlap in their genomic profiles. Both are characterised by amplifications in genes such as ERBB2, VEGFA, and GATA6, and exhibit high levels of aneuploidy, suggesting these cancers may represent a continuum rather than entirely distinct diseases [8, 10]. Notably, CIN gastric cancers and adenocarcinomas frequently co-cluster in integrative molecular analyses, whereas both are distinct from other subtypes like microsatellite instability (MSI) or Epstein–Barr virus (EBV)-associated gastric cancers [10].

Previous network meta-analyses (NMAs) combine data concerning oesophageal and GEJ cancers [11, 12] or GEJ and gastric cancers [13] since many trials use overlapping classification schemes. Others have addressed research questions focusing on treatment modalities for the best neoadjuvant treatment for gastric and gastrooesophageal adenocarcinomas [14] or oesophageal carcinomas [15], the efficacy and safety of first-line chemotherapy [16], and the efficacy of preoperative chemoradiotherapy and chemotherapy [17]. The comparison among multimodal treatments [18] was investigated in an NMA focused only on resectable GEJ carcinomas. An individual patient data (IPD) [19] meta-analysis was conducted to investigate the effect of neoadjuvant chemotherapy and chemoradiotherapy compared to surgery alone in patients with oesophageal and GEJ cancers. Although the above meta-analyses are thorough in their methodology and implementation, we target to investigate a broader spectrum of the disease and the available treatment modalities while also appraising the confidence in their results.

Therefore, this NMA protocol aims to gather all available data on oesophageal, GEJ, and gastric cancers, following PRISMA for NMA guidance [20]. A wider set of inclusion criteria will be required to accommodate the research question, and several different methodologies will be used to address challenges in synthesis. We will present a critical appraisal for the confidence in the results using CINeMA [21].

## Methods

This is a protocol for a network meta-analysis of randomised controlled trials comparing treatment modalities for oesophageal, gastroesophageal, and gastric cancer. This NMA protocol is registered in PROSPERO (CRD42025634169).

### Eligibility criteria

#### Types of participants

Eligible participants will be adults with diagnosed oesophageal, gastroesophageal, and gastric neoplasms at any stage or at recurrence, with or without prior treatment.

#### Types of interventions and comparators

##### Intervention(s), exposure(s)

Included interventions will be treatment modalities for oesophageal, gastroesophageal, and gastric neoplasms will include surgery, chemotherapy, chemoradiotherapy, immunotherapy, and additional therapies, including alternative therapies. Surgery will include all types of surgery, including submucosal excision. Palliative resections will not be excluded. All chemotherapeutic regimens, either neoadjuvant or adjuvant, will be eligible for comparison. Older agents will not be excluded. Any combination of chemotherapeutic agents and radiation, either neoadjuvant or adjuvant, will be included.

##### Comparators /controls

Under the scope of this systematic review, we aim to assess all available treatments within an NMA framework and as such, no control treatment will be defined. Two common comparators will be considered for the networks: surgery with any intention (treatment or palliative) and chemotherapy.

#### Outcomes

The primary outcome will be overall survival, defined as the length of time from diagnosis that patients with the disease are still alive. Secondary outcomes will include the metrics that follow. Progression-free survival, defined as the length of time from random assignment to any clinical trial to disease progression or death from any cause. Disease-specific survival, defined as the length of time from diagnosis that patients did not die due to the disease. Dropout rate, defined as the proportion of randomised patients that discontinued treatment for reasons related to it. Adverse effects, defined as the number of patients presenting with at least one adverse effect as defined in individual studies. The adverse effects will include hematologic disorders, nausea, and mucositis. If available, toxicity per event will be classified as proposed by NCI 2003 common toxicity criteria, in 5 classes: grade 0 (no toxicity), grade 1 (mild toxicity), grade 2 (moderate toxicity), grade 3 (severe toxicity), grade 4 (life-threatening toxicity), and grade 5 (death). Relapse, defined as the local, regional, oligometastatic, and distant recurrence-free proportions. Local recurrence is defined as recurrent disease isolated to the oesophageal lumen or conduit, with no evidence of any other regional or distant metastatic disease. Regional recurrence is defined as recurrent disease limited to the regional lymph nodes. Oligometastatic recurrence is defined as metastatic disease limited to up to five lesions that are safe to treat. Distant recurrence is defined as hematogenous metastasis to nonregional lymph nodes, visceral organs, bone, pleura, and/or peritoneum. Pattern of relapse was defined as the time until a local, regional, oligometastatic, or distant recurrence was first recorded. Patients who experienced distant recurrence/progression and local or regional recurrence/progression on the same date were counted as distant progression. R0 resection, defined as the proportion of patients in which an R0 resection was performed. Surgical mortality, defined as death occurring within 30 days from surgery. Operative morbidity, defined as the temporary or permanent disability observed during and after an operation. Quality of life will be recorded, if available, according to the scale and metrics used in the primary study.

#### Types of studies

Randomised controlled trials regardless of blinding will be included in this ΝΜΑ. Only reports with available full text will be included.

### Search strategy

Two medical databases (MEDLINE via PubMed and The Cochrane Library via CENTRAL) will be searched using the following terms:

- (esophagogastric OR oesophagogastric OR gastroesophageal OR cardia OR gastric OR stomach OR oesophagus OR esophagus OR oesophageal OR esophageal)
- AND
- (neoplasm OR cancer OR tumor OR adenocarcinoma OR carcinoma OR (squamous cell carcinoma))
- AND
- (chemotherapy OR chemoradiotherapy OR (adjuvant OR neoadjuvant OR perioperative) OR immunotherapy OR surg*)

### Selection of studies

We will set no restrictions for publication period. Full text reports in English, German, French, and Spanish will be considered for inclusion. We will use the clinical trial filter in PubMed to retrieve relative references. We will re-run the search prior to the final analysis. We will look up references from previous meta-analyses to ensure adequate retrieval of relevant reports. We will expand the search in clinical trial registries (namely ClinicalTrials.gov and International Clinical Trials Registry Platform (ICTRP) via Cochrane Central) to identify ongoing trials and we will attempt to retrieve results from unpublished trials. Two independent reviewers will be involved in screening and data extraction. We will use Covidence [22] to complete screening and data extraction.

### Data collection process

A data extraction form will be developed to collect various pieces of information for each study. It will include the report identity, consisting of the first author, year of publication, publication venue, DOI, registration, protocol number, funding, and the role of the funding source. In the study design section, we will record the number of sites, power analysis, interim analysis plan, and whether the statistical analysis plan was published. The form will also include information on population characteristics, such as age (in aggregate form), gender distribution (proportion of female/male participants), origin proportions (proportion of ethnic origin), WHO performance status, alcohol consumption (drinks per week or categorical), and smoking habits (pack years or dichotomous).

For condition characteristics, the form will include details on diagnosis, staging at the time of diagnosis, histopathological type, receptor status, lymph node status, metastatic status, mutations, and tumour location (epicentre if specified). In terms of compared treatments, we will collect data on experimental treatments (including components, dose, and time), comparator treatments (including components, dose, and time), and surgical approaches (open, minimally invasive, hybrid minimally invasive, and procedure).

The outcomes section will record overall survival with the corresponding hazard ratio and 95% confidence intervals for aggregate data. Additional survival metrics will be recorded including the number of patients surviving at 5 years, the number censored due to death or dropout, and the median survival time along with any metric of dispersion or confidence intervals. Toxicity will be recorded by the number of patients in each toxicity grade, while the dropout rate will capture the number of randomised patients who discontinued treatment for reasons related to the allocated modality. The form will document adverse effects across all available grades; the primary analysis will focus on severe and life-threatening adverse effects (grade ≥3), with the number of patients affected and, where available, the number of events per patient. Relapse data will include the number of patients with defined relapse types and details on patterns of relapse, including local, regional, oligometastatic, and distant recurrence-free survival, each with hazard ratios and 95% confidence intervals for aggregate data. Finally, the form will track R0 resection status (number of patients who underwent an R0 resection), surgical mortality (number of patients who died within 30 days of surgery), and operative morbidity (number of patients with temporary or permanent disability during and after the operation).

### Individual patient data request

Individual patient data will be sought by direct contact with study authors. To ensure every possible source for IPD will be sought after, an inquiry in the NIMH Data Archive will be submitted.

### Dealing with missing data

An attempt to obtain missing data from study authors is planned. Wherever possible, missing metrics will be calculated from those reported (i.e. missing standard deviations will be calculated from standard errors, ranges, or 95% confidence intervals) [23]. We will use cut-offs to decide for model implementation: more than 30% missing IPD or more than 50% missing data for covariates will halt the execution of the model.

### Assessment of methodological quality

RoB2 will be used to assess each study’s risk of bias. One reviewer will assess all studies across all domains of the tool and a second one will review the assessment. Discrepancies will be addressed by a third reviewer. Visualization tools will be used to summarize the assessment.

### Data synthesis and statistical analysis

Descriptive statistics of eligible trials, both for trial and sample characteristics, will be generated and reported. If the fundamental transitivity assumption is met, a network meta-analytical model should be appropriate to use.

### Pairwise meta-analysis

Pairwise meta-analysis will be performed for each direct comparison identified, including data from trials with all eligible anatomic locations and stages. Depending on the outcome, hazard ratios (survival outcomes) or risk ratios (dichotomous outcomes, like adverse events) along with 95% confidence intervals will be calculated and synthesized in a random-effects model. The restricted maximum likelihood estimator will be used for the heterogeneity variance. The Knapp-Hartung-Sidik-Jonkman method will be used to estimate 95% confidence intervals for the overall effects. Statistical heterogeneity will be assessed by visual inspection of the resulting forest plots as well as the tau-square and the I2 statistics and prediction intervals [24]. A summary of findings (SoF) table will be constructed. The meta library for R [25] will be used for calculations.

### Evaluation of transitivity

Transitivity will initially be investigated by comparing the study and sample characteristics to evaluate whether participants are in principle eligible for all available interventions and it is sensible to assume that they are jointly randomisable [22]. All available direct comparisons will then be assessed for their homogeneity with respect to the distribution of potential effect modifiers. All factors that could be modifying the measured effect will be described and their distributions will be plotted across different comparisons using box plots. Candidate factors will be age, origin, diagnosis, stage at the time of diagnosis, alcohol consumption, and smoking.

### Network meta-analysis

The primary goal is to conduct an inclusive NMA accounting for all available information from all disease stages and locations. Networks of interventions will be formed for synthesis for each outcome, if the transitivity assumption is likely to hold. A random-effects NMA model will be fit to simultaneously compare all treatments in the network. We will fit NMA using the graph theoretical approach as described by Rücker [26]. Treatments will be ranked using P-scores as proposed by Rücker and Schwarzer [27]. P-scores are the frequentist analogue to surface under the cumulative ranking curve and are derived from the probability that an absolute difference of the observed size or larger occurs, given a null hypothesis of no difference is true [27]. Their interpretation can be extended as the average extent of certainty that a treatment is better than all other competing treatments and as a result the P-score represents the rank of each treatment. Values closest to 1 mean theoretically better and closest to 0, worse. Network plots will be used to schematically represent the available treatment comparisons. Nodes will represent the treatments and edges will correspond to direct comparisons from eligible studies. The size of nodes will be proportional to the number of participants allocated to them and the thickness of the edges will represent the number of included studies directly assessing the comparison. League tables will be used to report the NMA estimates among all possible pairwise comparisons and their confidence intervals. The netmeta [28] and the multinma [29] libraries for R will be used for calculations.

Two additional meta-analytic models will be implemented:

- Population-adjusted methods with the retrievable individual patient data will be implemented to accommodate for potential differences across populations of individual trials [30]. Treatment effects for each type of population will be calculated within the NMA model.
- Component NMA will be used to address treatment lumping and explore the added benefit of treatment modalities’ combinations. Multimodal treatment is frequent in this population, based on clinical practice guidelines, and their separate effect has yet to be addressed [31, 32]. The components under consideration will be chemotherapy regimens (neoadjuvant or adjuvant), surgery, radiation protocol, immunotherapy, and synergic therapies, if identified in individual studies.

### Assessment of coherence

Coherence will be examined both with global and local methods. Standard methodology will be applied for global coherence of the entire network using the design-by-treatment interaction mode [33]. The SIDE (Separating Indirect from Direct Evidence) [34] approach will be implemented to evaluate local coherence between direct and indirect evidence for each pairwise treatment comparison.

### Subgroup analyses

Subgroup analysis is planned both for pairwise meta-analysis and NMA. Three factors will be considered: diagnosis, tumour location, and stage at diagnosis:

1. Diagnosis will be classified according to the pathology reports (adenocarcinoma, squamous cell carcinoma).
2. Tumour location will be classified according to primary tumour epicentre, according to the 8th AJCC [35]:
  - Thoracic oesophagus
  - Abdominal oesophagus (including tumours involving the gastroesophageal junction with epicentre no more than 2 cm into the proximal stomach)
  - Gastric cancers (tumours with epicentre located greater than 2 cm into the proximal stomach are staged as stomach cancers even if GEJ involved)
3. Stage at diagnosis will be defined with the 8th edition AJCC criteria into four categories (I–IV).

### Meta-regression analyses

Network meta-regression will be used to explore candidate factors of effect modification: age, gender, ethnic origin, and tumour biology, and investigate whether statistical heterogeneity and/or incoherence are reduced. Each described covariate will be assessed in a different meta-regression model using two modelling assumptions: (a) a common coefficient across all comparisons, considering surgery as reference treatment, and (b) comparison-specific and consistent coefficients. Depending on the number of studies, multivariable models with interactions between covariates will be considered.

### Small-study effects and publication bias

Publication bias will be assessed by visual inspection of funnel plots [36], comparison adjusted funnel plots [37], and Egger’s test if there are at least 10 studies per comparison for the funnel plots and in total for the comparison adjusted funnel plot. These methods test for small-study effects, which we will use as a proxy for publication bias.

### Quality of evidence

The CINeMA framework will be used to assess the confidence in the results of the NMA. The six domains will be assessed at three levels (no concerns; some concerns; major concerns): within-study bias, reporting bias, indirectness, imprecision, heterogeneity, and incoherence. The range of equivalence used for the judgements of imprecision, heterogeneity, and incoherence will be defined from previous meta-analyses focusing on overall survival. Within-study bias will be derived from the RoB2 assessment.

### Ethics and dissemination

An application was submitted to the Ethics Committee of the Aristotle University of Thessaloniki and received approval with number 130/2025. A paper will be submitted in a peer-reviewed journal to be assessed for publication. The primary aim of this review is to address the research question outlined above; additionally, the protocol and the resulting dataset are expected to serve as an illustrative example for meta-research methodology development.

### Funding

There is no funding for this meta-analysis.

## Discussion

This NMA aims to investigate the effect of available treatment modalities in patients with oesophageal, GEJ, or gastric carcinomas. Identifying these entities’ underlying patterns concerning their response to the treatment strategies might reveal new insights on their shared characteristics and clinical progression. Existing meta-analyses address a narrower scope of criteria concerning either population, comparator, or outcomes.

The most prominent strength of the proposed protocol is the implementation of existing meta-analytical models that will allow the assessment of the extended inclusion criteria. Though no simultaneous assessment of all the extended criteria is currently feasible, the suggested approaches are expected to yield summative results that will capture this wider knowledge base and identify the parameters that should be taken into consideration before attempting to synthesise data for more inclusive research questions. Additionally, the implementation of the CINeMA approach for the outcomes will provide the first, to our knowledge, formal assessment of the confidence in the results of NMA on the subject.

While we are aware of the potential heterogeneity that might be present, population-based evidence further reinforces the decision to pool these cancers for analysis. A large cohort study by Pape et al. [38] compared advanced oesophageal, GEJ, and gastric adenocarcinomas, finding no significant differences in overall survival among these groups after adjusting for clinical and demographic factors. Despite variations in clinical presentation, such as metastatic burden and treatment patterns, survival outcomes were comparable. These findings suggest that oesophageal, GEJ, and gastric cancers can be included together in clinical trials and analyses, as they may benefit from shared therapeutic approaches and overlapping biological characteristics. From a therapeutic perspective, these cancers share common molecular targets, such as HER2 and VEGFA amplifications, as well as responsiveness to immune checkpoint inhibitors targeting PD-1/PD-L1 pathways. Clinical trials and submitted clinical trial protocols increasingly include all three cancer locations in evaluating novel treatments, recognizing their overlapping biology and similar treatment paradigms [9]. Pooling data will allow for increased statistical power and a broader understanding of the efficacy of treatment strategies across these related cancers.

## Data Availability

The datasets used and/or analysed during the current study are available from the corresponding author on reasonable request.

## Abbreviations

GEJ: Gastroesophageal junction
NMA: Network meta-analysis
RCTs: Randomized controlled trials
IPD: Individual patient data
CIN: Chromosomally unstable subtype of gastric adenocarcinomas
MSI: Microsatellite instability
EBV: Epstein–Barr virus
SoF: Summary of findings
